# Some, but not all of the premenstrual syndrome symptoms affect the medical exam scores in medical students

**DOI:** 10.12669/pjms.37.4.3931

**Published:** 2021

**Authors:** Filiz Bilir, Ramazan Akdemir, Cemil Bilir

**Affiliations:** 1 Filiz Bilir, Department of Medical Education, Sakarya University School of Medicine, Sakarya, Turkey; 2 Ramazan Akdemir, Department of Cardiology and Medical Education, Sakarya University School of Medicine, Sakarya, Turkey; 3 Cemil Bilir, Department of Internal Medicine, Sakarya University School of Medicine, Sakarya, Turkey

**Keywords:** Premenstrual syndrome symptoms, Medical students, Exam scores

## Abstract

**Objectives:**

This research aims to identify the effects of premenstrual syndrome (PMS) symptoms on the school exam scores in medical students.

**Methods:**

This cross-sectional study was designed at Sakarya University School of Medicine The study included medical students who were in the first, second, and third year of class. In this study, there were 193 male and 100 female students. The study investigated how PMS symptoms affected medical student’s exam scores and school success. All exam scores were recorded during the two-consecutive semester so duration of study was one year

**Results:**

There were 100 female students, and they had five different committee exams for one year. Female student’s exam scores were significantly higher for four committees and an average score of all year. The mean age of female students was 19.9 ±1.5. Acne, nausea/vomiting, sleeping, abdominal bloating, and prurience change had significantly different exam scores compared to the group without these symptoms. Students with acne had substantially higher exam scores than without acne; inversely, the other four symptoms negatively affected exam scores.

**Conclusion:**

Some of the PMS symptoms can be more annoying and should change the quality of life more than the other symptoms, so we should define these symptoms to improve our student’s quality of life and school success.

## INTRODUCTION

Menstrual cycle has two main phases, one is an ovarian cycle, and the second one is uterine cycle. In the ovarian cycle there are two phases and first one is estrogen dominant called as follicular phase and the second is progesterone dominant period called as luteal phase. These hormonal changes should effect women metabolism as well as quality of life.^1^

Premenstrual syndrome (PMS) is defined as a woman in reproductive age. It has emotional or physical symptoms that start a couple of days before the menses, ameliorate after the menstrual cycle.^2^ PMS’s most common symptoms are nausea, vomiting, thirsty, vertigo, depression, mood changes, dizziness, flatulence, mastalgia, crying, fatigue, irritability, anxiety, low concentration, and forgetfulness, appetite changes, gastrointestinal symptoms, and edema.^3^ Indeed there are no exact criteria for the diagnosis because of the subjectivity of PMS symptoms, ambiguous complaints, and psychological components of these symptoms make the diagnosis hard. Determining the symptoms time and at least one effective symptom with one somatic symptom, which causes a dysfunction in work, education, or social performance are essential.^4^ Therefore, criteria of the American College of Obstetricians and Gynecologists’ are used for diagnosis.^5^ The physical and emotional symptoms in PMS can decrease women’s quality of life (QOL), but there are conflicting results with the effect of PMS symptoms on QOL.^6^,^7^

PMS affects regular activities and social skills and quality of life and social skills.^8^ Also, the severity of PMS symptoms can change with their duration and progress to the Premenstrual dysphoric disorder (PMDD); additionally, these menstrual changes, particularly in the luteal phase, are related to many cognitive, psychological, and behavioral changes.^9^ PMS and PMDD affect reproductive ages, socially and psychologically active adults, but we don’t know how these findings influence academic performance. Limited data about academic life have been presented previously and most of them had investigated the relationship between quality of life and PMS or PMDD. In a Brazil study included 642 students that found 49.9% of PMS and 23.3% of PMDD prevalence in addition they found close relation with lower quality of life and severity of PMS symptoms. However, a study published by Antunes et al. included university students in the same population did not find decreased PMDD symptoms in students who were using combined oral contraceptive pills.^10^,^11^ On the other hand it is well known that students with good physical health have better academic performance but, there is no data about medical students who had PMS symptoms and their exam scores.^12^ That’s why this study aimed to investigate the possible relationship between PMS symptoms and the medical students’ exam success.

## METHODS

This cross-sectional study was designed at Sakarya University School of Medicine after the approval of the local ethical committee (Ref: 71522473/050.01.04/135, Dated: 07/05/2019). The study included medical students who were in the first, second, and third year of class. Between the two semesters, all medical student exams scores were recorded. They completed five different exams during the two semesters so duration of study was one year. A gynecologist and obstetrician researcher examined and then filled the students’ data such as abdominal ultrasound findings, medications history, physical or emotional complaints and exam scores during their free time and the PMS form. Participation in the study was voluntary.

### Inclusion criteria

It included PMS symptoms for instance prurience, anxiety, irritability, depression symptoms, social withdrawal, tenderness of breast, abdominal bloating, headache, edema in addition to PMS symptoms also normal abdominal ultrasound findings, and normal ovulatory functions were the other inclusion criteria.

###  Exclusion criteria

Amenorrhea, endometriosis, pregnancy, and chronic disease, causing abdominal pain, using oral contraceptive drugs or IUD, and students with adnexal mass or myoma uteri USG. The demographic form questions included lifestyle and reproductive variables, economic status, age, smoking history, and menstrual characteristics. The symptom dates were constructed based on ACOG PMS criteria, which included behavioral and somatic symptoms as mentioned above. The diagnosis was accepted as PMS if a female student had at least one mood and one physical symptom.

All data analyses were done with SPSS version 18.0 (SPSS Inc., Chicago, IL). Descriptive statistics described the sample and main variables such as means and standard deviation, and frequency. Qualitative variables were analyzed with the chi-square test and quantitative data with the Student t-test. Spearman’s rho correlation was used to define the relationship between the variables. Regression analysis used for exploring the predictability of students’ exam scores based on different variables and P values lower than 0.05 was considered statistically significant.

## RESULTS

**Fig.1:**
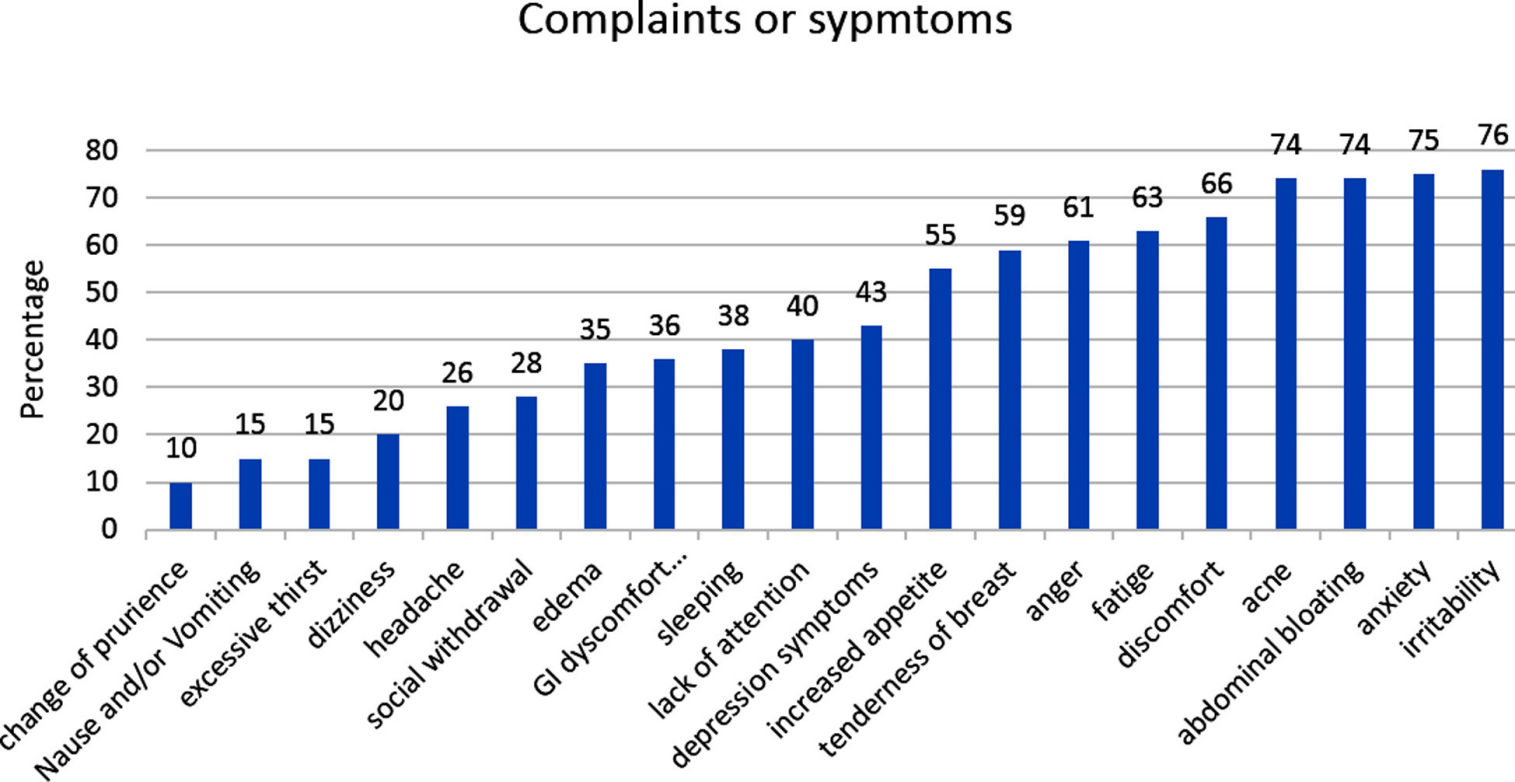
Percentage of each symptom or complaint in female students.

In this study, there were 193 male and 100 female students. In one year, students had five different committee exams, and we compared five of these exams and average scores, both male and female (Table-I). Female student’s exam scores were significantly higher for four committees and the average score of all year. The mean age of female students was 19.9 ±1.5, body mass index was 22, and weight was 58,9. We examined 20 different complaints or symptoms with a questionnaire and physical examination and summarized in Fig.1. The most common four findings were acne, abdominal bloating, anxiety, and irritability.

**Table-I:** Male and Female Exam Scores.

Exam Score	Male (SD)	Female (SD)	P value
1st committee	65.9 (13)	70.2 (11)	0.007
2nd committee	61.7 (19)	67.4 (12)	0.015
3rd committee	66.3 (16)	71.3 (14)	0.008
4th committee	66.1 (17)	73.5 (13)	0.0001
5th committee	71.8 (16)	75 (16)	0.058
Average score	67.3 (14)	73 (13.7)	0.001

Acne, nausea/vomiting, sleeping, abdominal bloating, and prurience change had significantly different exam scores compared to the group without these symptoms. Abdominal bloating was one of the most common symptoms of our study population. Seventy-four students had abdominal bloating, and the remaining 26 did not besides the average exam scores were 71.6 vs. 77, respectively (p< 0.05). Also 3^rd^, 4^th^ and 5^th^ committee scores were significantly lower in students who had abdominal bloating (69.5 vs 76.3 with P-value 0.03, 72 vs 77.8 with P-value 0.05 and 73 vs 81.7 with P-value 0.018, respectively). Acne is the 4^th^ most common symptom; Seventy-four students had acne and 26 did not. Exam scores was 74.8 in the group who had acne and it was 68.9 in the group who did not have acne and it was statically significant (p< 0.05). According to the end-of-year average exam scores sleepy and nausea/vomiting were the other two significant factors that affect the exam scores. Students who were sleepy had significantly lower exam scores as well as nausea/vomiting, 69 vs. 75, and 65 vs. 74, respectively. All significant values are presented in Table-II.

**Table-II:** Exam scores and premenstrual syndrome symptoms.

Exam	Acne Yes/no	Nause/ vomiting Yes/no	Abd.bloating Yes/no	Sleeping Yes/no	Change of prurience Yes/no
1st Committee	72 vs 64 P:0.003	64.9 vs 71 P:0.052	70 vs 70 P:0.9	68 vs 71.6 P:0.12	62 vs 71 P:0.018
2nd Committee	68 vs 62 P:0.08	62 vs 68 P:0.2	66 vs 70.8 P:0.17	64 vs 68.8 P:0.2	59 vs 68 P:0.08
3rd Committee	72 vs 68 P:0.22	61 vs 73 P:0.004	69.5 vs 76 P:0.033	68 vs 73 P:0.07	61.9 vs 72 P:0.026
4th Committee	75 vs 68 P:0.032	79 vs 77 P:0.2	72 vs 77.8 P:0.054	69 vs 75.9 P:0.019	65 vs 74 P:0.04
5th Committee	77 vs 70 P:0.03	64 vs 77 P:0.003	73 vs 81 P:0.018	70 vs 78.7 P:0.01	72 vs 75.9 P:0.5
Average Score	74 vs 67 P:0.012	65 vs 74 P:0.009	71 vs 77 P:0.048	69 vs 75 P:0.016	66 vs 73 P:0.07

## DISCUSSION

In this study, we found that five of the PMS sign and symptoms such as acne, nausea/vomiting, changes in sleep pattern, abdominal bloating, and change of prurience affected the school exam scores and academic performance. Symptoms included nausea/vomiting, change in sleep pattern , abdominal bloating, and change of prurience caused lower exam scores than without these symptoms on the other hand students who had acne had higher exam scores. This is the first study that investigated how PMS symptoms affect the academic performance of medical students.

The prevalence of PMS in the Turkish population was reported between the ranges of 6.1%-91.8%. This prevalence can be changed according to age or population characteristics; for example, it was 37% in nursing students and 72% in medical school students.^13^,^14^ All these studies have focused on severity of symptoms and effects on quality of life however they did not investigated the academic success or how those symptoms change school life. Also, different population studies showed a broad range of 24%-100%.^15^,^16^ Furthermore, only a few studies on medical students found a variation in PMS prevalence, roughly 90% in Egypt with ICD-10 criteria, 35% in Saudi Arabia used the ACOG criteria, and 51% in Pakistan according to ICD-10.^17^,^18^ Our results are also concordant with the literature and the PMS prevalence was around 60%. Goker et al. found the most common symptom was abdominal bloating with 88% and irritability as an emotional symptom around 88%.^7^ A different population study, which included Nigerian university students published by Adewuya et al., showed that breast tenderness was 35.5%. A second study found that breast tenderness was the most frequent symptom in Lebanese medical students, and the rate was 65%.^3^,^9^,^19^ Similar to these results, our study found abdominal bloating, anxiety, and irritability were the three most common symptoms, and breast tenderness was around 60%.

We also know that younger women have more severe and significantly more symptoms, so this can explain why PMS decreased the quality of life in younger ages.^19^ More than 70% of students had at least one PMS symptom in our study, but until this study, we did not know which symptoms can significantly affect the quality of life, especially medical students. A study showed a significant correlation between the general health score SF-36 with PMS and severe PMS patients had the lowest SF-36 scores. In the same study, the authors found that the lowest mean SF-36 score was correlated with emotional functions (Social withdrawal, angry outbursts, anxiety, and depression) in the severe PMS group.^7^ Taghizadeh et al. found that “the more the severity of PMS, the less the quality of life in mental health,” and it can cause depression, nervousness, and sadness.^20^ Nisar et al. investigated PMS in medical students. They found that quality of life score was significantly lower in the PMS, and many symptoms such as emotional problems, physical problems, vitality, mental health, and body pain significantly differ. SF-36 scores were significantly lower in the group who had PMS compared to those without PMS.^17^ One of the studies investigated PMS symptoms on how to affect the quality of life, even they found significant deterioration of quality of life, but they did not explore the school success or exam scores of students. Our study compared 20 different symptoms with exam scores, and only four of them were significantly different for the end of year scores. Students who had acne had significantly higher exam scores, especially the first, 4^th,^ and 5^th^ committee and the end of the year exam score. On the other hand, the other four symptoms, nausea/vomiting, change in sleep pattern, abdominal bloating, change of prurience affect negatively, and students who had these four symptoms had significantly lower exam scores.

A Turkish college student study revealed that the PMS rate was 72% in college students, which adversely affects the quality of life, especially in the physical, mental, and environmental fields. Also, an Iranian study was carried out in medical students; authors found that mental health and environmental symptoms were significantly different in students who had severe PMS compared to without PMS symptoms.^21^,^22^ To our knowledge, our research is the first study that investigated what kind of PMS symptoms affect the quality of life in medical students in the field of school success. Hussein Shehadeh et al. investigated university students’ academic performance as GPA scores in premenstrual syndrome and premenstrual dysphoric disorder, and there was no significant difference between the groups. Still, they found that PMS prevalence was 92.3%, and PMDD was 7.7% among female university students.^23^ On the other hand, a different study investigated the PMS and exam scores in female university students. Like the previous research, they did not find a difference between the group with and without PMS student’s exam scores. Students who had a longer duration of menses (<21 days vs. 21-35 days vs.>35 days) had significantly lower exam scores.^24^ This finding has not reverberated to our results, but a longer menses period can decrease the study time, so that’s why they had lower exam scores.

During the follicular phase, estrogen begins to increase and it can affect the brain functions and change the female’s affective and cognitive status so that these interactions can be responsible for mood changes and cognitive functions. Therefore, fluctuating mood and cognitive skills can be a reason for these hormonal changes, especially in estrogen surge. Even we cannot say the exact mechanism of our results, we can speculate this, increasing serum testosterone levels should be related to premenstrual acne, and testosterone has neuroprotective effects. It can improve memory and verbal learning.^25^ In our study, students with acne had significantly higher exam scores. Students with decreased prurience had lower exam scores; these two clinical findings can be related to the fluctuation of serum testosterone levels during the menses. The treatment of these symptoms should increase students’ quality of life and may improve our female student’s academic success.

### Limitations of the Study

We included students who had PMS symptoms, not focused on PMS diagnosis, so some of the students could diagnose PMS and PMS symptoms. Additionally, many other reasons could affect the exam scores, and to exclude these factors, we compared the exam scores with males and females.

## CONCLUSION

In conclusion, some of the PMS symptoms had a higher clinical impact. They can change the quality of life more than the other symptoms. If we define these symptoms clearly, such as acne has a positive effect, nausea/vomiting, change in sleep pattern, abdominal bloating, and change of prurience has a negative impact.

### Note

The study participants given their written informed consent and that the study protocol was approved by the institute’s committee on human research.

### Authors’ Contributions

**FB:** Study design and gynecologic examination of all students. Responsible and accountable for the accuracy or integrity of the work.

**RA:** Editing the manuscript.

**CB:** Collecting data and analysis of them.
